# Glucose Transporter Glut1-Dependent Metabolic Reprogramming Regulates Lipopolysaccharide-Induced Inflammation in RAW264.7 Macrophages

**DOI:** 10.3390/biom13050770

**Published:** 2023-04-29

**Authors:** Alex Cornwell, Hubert Ziółkowski, Alireza Badiei

**Affiliations:** 1 Department of Biology and Wildlife, College of Natural Science and Mathematics, University of Alaska Fairbanks, Fairbanks, AK 99775, USA; 2 Department of Pharmacology and Toxicology, Faculty of Veterinary Medicine, University of Warmia and Mazury in Olsztyn, Oczapowskiego 13, 10-718 Olsztyn, Poland; 3 Department of Veterinary Medicine, College of Natural Science and Mathematics, University of Alaska Fairbanks, Fairbanks, AK 99775, USA

**Keywords:** macrophage, Glut 1, inflammation, autophagy, hydrogen sulfide

## Abstract

This study investigated the critical role of Glut1-mediated glucose metabolism in the inflammatory response of macrophages, which are energy-intensive cells within the innate immune system. Inflammation leads to increased Glut1 expression, ensuring sufficient glucose uptake to support macrophage functions. We demonstrated that using siRNA to knock down Glut1 reduces the expression of various pro-inflammatory cytokines and markers, such as IL-6, iNOS, MHC II/CD40, reactive oxygen species, and the hydrogen sulfide (H_2_S)-producing enzyme cystathionine γ-lyase (CSE). Glut1 activates a pro-inflammatory profile through a nuclear factor (NF)-κB, while silencing Glut1 can prevent lipopolysaccharide (LPS)-induced IκB degradation, blocking NF-κB activation. Glut1’s role in autophagy, an essential process for macrophage functions such as antigen presentation, phagocytosis, and cytokine secretion, was also measured. The findings show that LPS stimulation decreases autophagosome formation, but Glut1 knockdown reverses this effect, increasing autophagy beyond control levels. The study highlights Glut1’s importance in macrophage immune responses and its regulation of apoptosis during LPS stimulation. Knocking down Glut1 negatively impacts cell viability and mitochondrial intrinsic pathway signaling. These findings collectively suggest that targeting macrophage glucose metabolism through Glut1 could potentially serve as a target for controlling inflammation.

## 1. Introduction

At the outset of microbial invasion, immune cells migrate into infected tissues, triggering a cascade of innate defenses. These defenses include the generation of reactive oxygen species (ROS) and the activation of transcription factors, which in turn regulate the expression of proinflammatory mediators. The indispensable role of macrophages in coordinating these responses underscores their crucial role in initiating and sustaining both innate and adaptive immune responses, and ultimately restoring homeostasis [1]. Emerging evidence suggests that glucose substrate availability exerts regulatory control over macrophage activation, and subsequently impacts their antimicrobial activity. Moreover, in certain pathological conditions such as atherosclerosis, glucose plays a direct role in promoting macrophage activation, thereby driving disease progression [2]. However, the role of glucose metabolism in influencing macrophage proinflammatory innate responses, as well as adaptive immune interface responses is still not fully understood.

As blood monocytes migrate to injured tissues during infection, they undergo a maturation process that is accompanied by a progressive increase in major histocompatibility (MHC) II expression [3]. Phagocytic activity is stimulated by microbes or damaged tissues binding to phagocytic receptors [4]. Stimulation of surface Toll-like receptors (TLRs) trigger the expression of proinflammatory cytokines, such as Interleukin (IL) 6, IL-1β, and tumor necrosis factor alpha (TNF), among others [3]. Intracellular mechanisms are in place to precisely regulate cytokine secretion and antigen presentation. Among these, autophagy plays a key role in controlling immune receptor expression and the secretion of inflammatory mediators, highlighting its importance in macrophage function [4], and is necessary for the continued presentation of antigens on MHC II to improve helper T cell stimulation [5]. We hypothesize that glucose is important in these processes in macrophages. Given the preferential use of glycolysis in proinflammatory macrophages [6], we postulate that glucose is a critical factor in regulating their function, with the glucose transporter 1 (Glut1) playing a pivotal role as the primary rate-limiting glucose transporter in these cells [2,7].

The aim of this study is to investigate the role of Glut1-mediated glucose metabolism in the proinflammatory response of macrophages by knocking down its expression using siRNAs. The study seeks to determine the impact of Glut1 inhibition on proinflammatory cytokine production, MHC II surface expression, ROS levels, and iNOS expression. Additionally, the study aims to explore the association between Glut1 inhibition and autophagy, as well as its impact on macrophage survival and apoptosis. The ultimate goal is to identify Glut1 as a critical driver of the innate and adaptive immune response and a potential therapeutic target for inflammatory diseases.

## 2. Materials and Methods

### 2.1. Macrophage Cell Culture

RAW264.7 (ATCC, Manassas, VA, USA) murine macrophage cell lines were cultured in DMEM (Gibco, Waltham, MA, USA) containing 10% (*v*/*v*) heat-inactivated fetal bovine serum (FBS; Cell Applications Inc., San Diego, CA, USA), 100 units/mL penicillin, and 100 μg/mL streptomycin (Gibco) and maintained at 37 °C in a humidified atmosphere containing 5% CO_2_. Cells were counted to seed 2.4 × 10^6^ cells on a 6-well culture plate and grown to confluence. After reaching 70% confluence, macrophages were ready for treatment.

### 2.2. Macrophage Treatment with Lipopolysaccharide (LPS)

Macrophages were stimulated with *E. coli*-derived LPS (100 ng/mL; Invitrogen, Waltham, MA, USA) for 4 h or the indicated times. The concentration of LPS has been reported by other laboratories to induce immune and pro-inflammatory responses in macrophages [8,9,10,11]. After treatment, macrophages were removed from the plates for the preparation of flow cytometric staining or extraction of RNA or protein.

### 2.3. Trypan Blue Cell Count

Cell counts were determined with the trypan blue exclusion assay immediately after treatments. Briefly, an aliquot (10 μL) of the cell suspension is diluted 1:1 (*v*/*v*) with 0.4% trypan blue (Thermo Fisher, Waltham, MA, USA) and viable cells are counted with a hemocytometer (Countess 3, Thermo Fisher).

### 2.4. Protein Extraction and Western Blot

The treated macrophage cells were washed with ice-cold PBS and then lysed in RIPA cell lysis buffer, and a 1% Halt protease inhibitor cocktail (Thermo Fisher, Waltham, MA, USA). The resulting cell lysates were centrifuged for 20 min. at 20,000 g at 4 °C, and the protein concentrations in the supernatants were determined using a Pierce BCA protein assay kit (Thermo Fisher, Waltham, MA, USA) [12]. 20 μg proteins were loaded onto 10% SDS-PAGE gels, followed by electro-transfer onto nitrocellulose-membrane (Bio-Rad). The membranes were blocked in 1  ×  TBST (0.1% Tween-20, 20 mM Tris–Cl (pH 8.0), and 150 mM NaCl) containing 5% nonfat dry milk powder and then incubated with the primary antibodies against Glut1 (1:1000 dilution, 66,290, Proteintech Rosemont, IL, USA), IL-6 (21,865, Proteintech Rosemont, IL, USA), IkBα (10,268, Proteintech, Rosemont, IL, USA), CSE (12,217, Proteintech, Rosemont, IL, USA), iNOS (22,226, Proteintech, Rosemont, IL, USA), and GAPDH (1:1000 dilution, 60,004, Proteintech, Rosemont, IL, USA) overnight at 4 °C. Membranes were washed 3 times (1 × TBST), and incubated with appropriate horseradish peroxidase-conjugated secondary antibodies (1:10,000, Proteintech, Rosemont, IL, USA) for 1 h at room temperature and then washed 3 times (1  ×  TBST). Lastly, immunoreactive proteins were detected using an enhanced chemiluminescence detection kit (Bio-Rad, Hercules, CA, USA). The band density was quantified by Image J 1.8.0172 software (National Institutes of Health NIH) and the representative data were experiment normalized to nontreated control.

### 2.5. RNA Extraction and RT-qPCR

Total RNA from cells was extracted using Trizol and chloroform reagents (Invitrogen, Waltham, MA, USA) following the manufacturer’s instructions. Sample concentrations were determined using Nanodrop One (Thermo Fisher, Waltham, MA, USA). First-strand cDNA synthesis was performed on 5 µg total RNA using M-MLV reverse transcriptase (Invitrogen, Waltham, MA, USA) and random hexamers (IDT, Coralville, IA, USA) and stored at −20 °C. PowerUp SYBR Green Mix (Applied Biosystems, Waltham, MA, USA) was used according to the manufacturer’s instructions in a 384-well format. To compare the mRNA levels between different samples relative expression versus control was reported [13], and data were normalized to GAPDH. Experiments were run in triplicate; each sample represents at least three technical repeats. The sense and antisense primers were designed to span Exon/Intron junctions using primer BLAST (NIH) and are shown in Table 1.

### 2.6. siRNA-Mediated Knockdown of Glut1 Gene

Silencer Select pre-designed siRNAs (Ambion, Austin, TX, USA) targeting the Glut1 gene and negative control siRNA were used in the gene silencing experiments. Following manufacturer instructions for lipofectamine RNAiMAX (Invitrogen, Waltham, MA, USA), cells were incubated with 5 pmol siRNA-lipofectamine complex for 24 h or indicated times. After incubation, the medium was replaced, and cells were further treated.

### 2.7. Low Glucose Treatment

For low glucose experiments, RAW264.7 cell line was either cultured in 1 g/mL low glucose DMEM (Gibco, Waltham, MA, USA) with added 3 g/mL glucose (normal glucose group) or 3 g/mL mannitol (low glucose group) for 24 h. Osmotic control was assured by treating RAW cells with equimolar concentrations of mannitol (Sigma-Aldrich, St. Louis, MO, USA). LPS was administered to cells following 24 h exposure to specified media. Following all treatments, RNA was extracted and analyzed as previously described.

### 2.8. Flow Cytometry to Measure MHCII, CD40, and CD80 Surface Levels

To stimulate MHCII, CD40, and CD80 expression, siRNA-treated macrophages were treated with 100 ng/mL LPS for 24 h. For immunofluorescence surface staining of macrophages, cells were suspended and fixed at a concentration of 10^6^ cells per 100 μL in 4% paraformaldehyde (PFA) for 15 min at room temperature. The cells were then washed with 1 × PBS (3 times) and suspended (10^6^ cells per 100 μL) in an antibody dilution buffer (1 × PBS containing 3% bovine serum albumin) with primary antibodies targeting MHCII for 1 h at 4 °C. After that the cells were washed 3 times with 1 × PBS followed by incubation with R-phycoerythrin R-PE (P-2771MP, Invitrogen), a conjugated secondary antibody for 1 h at 4 °C. For CD40 (65,062, Proteintech, Rosemont, IL, USA) and CD80 (65,076, Proteintech, Rosemont, IL, USA), R-PE conjugated primary antibodies were used. Finally, the cells were washed 3 times with 1 × PBS and resuspended in 1 × PBS containing 3% bovine serum albumin and immunofluorescence was detected by flow cytometry (Guava MUSE cell analyzer) using the 532 nm laser line with 576/7 nm dichroic filter. Gating to select for positive expression was set using IgG PE isotype controls. Data files were analyzed for percent positive by FlowJo software (Becton Dickson, Ashland, Oregon).

### 2.9. Flow Cytometry to Measure Annexin V and 7-AAD

For apoptosis detection assay staining of macrophages, cells were suspended (10^6^ cells per 100 μL) in buffer (1 × PBS containing 3% bovine serum albumin) and diluted in 2 × buffer containing Annexin V surface stain and 7-AAD DNA stain dyes (Millipore, Burlington, MA, USA) and kept at 4 °C. The cells were then immediately read by flow cytometry (Guava MUSE cell analyzer). Data files were analyzed for percent positive using negative (live cell) controls by floreada.io software.

### 2.10. Glutathione Level Assay

GSH level in macrophages was determined using a one-step fluorometric kit (Fluorometric-Green, ab13,8881, Abcam, Cambridge, UK) according to the manufacturer’s protocol. Potassium phosphate EDTA buffer (KPE) was prepared immediately before experiments [14]. Following treatments, macrophages were removed from culture plates and counted to 10^6^ cells per aliquot. Aliquoted cells were lysed in 0.5 mL KPE buffer containing 0.1% Triton X-100 and 0.6% sulfosalicylic acid and kept on ice. Cell lysates were then vortexed for 15 s and centrifuged at 8000 *g* for 10 min. The supernatants were transferred to pre-chilled microcentrifuge tubes. The concentration of total protein in each sample was determined by the Pierce BCA protein assay kit. Samples were then mixed 1:1 with glutathione detection reagent to a final volume of 100 µL on 96-well plates and incubated in the dark at room temperature for 30 min. Then, fluorescence intensity was monitored at EX/EM of 490/520 nm.

### 2.11. Autophagy Detection

The autophagy level in macrophages was determined using a fluorometric kit (Autophagy assay kit, MAK138, Millipore) according to the manufacturer’s protocol. Pre-siRNA-silenced cells were removed from culture dishes and plated in 96-well plates (10^4^ cells/well) and then treated with or without LPS overnight for 12 h. After incubation the growth medium was removed, the cells were washed and the autophagosome detection reagent working solution was added at a volume of 100 μL per well and incubated for 30 min in the dark at 37 °C. The cells were washed four times with a wash buffer. Fluorescence intensity measurements were conducted on a Biotek microplate reader using EX/EM of 355/538 nm.

### 2.12. Statistical Analysis

Statistical differences between experimental groups were determined using statistics software within GraphPad Prism (GraphPad Software, Inc., La Jolla, CA, USA). For all experiments, results are reported for at least *n* = 3. Data are expressed as mean ± SD. Statistical significance was determined by unpaired Student’s t-test for comparing two groups, and one-way analysis of variance with Tukey’s post hoc analysis was used for comparing three or more groups. *p*  ≤  0.05 was considered statistically significant.

## 3. Results

### 3.1. Glut1-Driven Glucose Uptake Drives NF-κB Mediated Pro-Inflammatory Profile in LPS-Treated Macrophages

**Figure 1:**
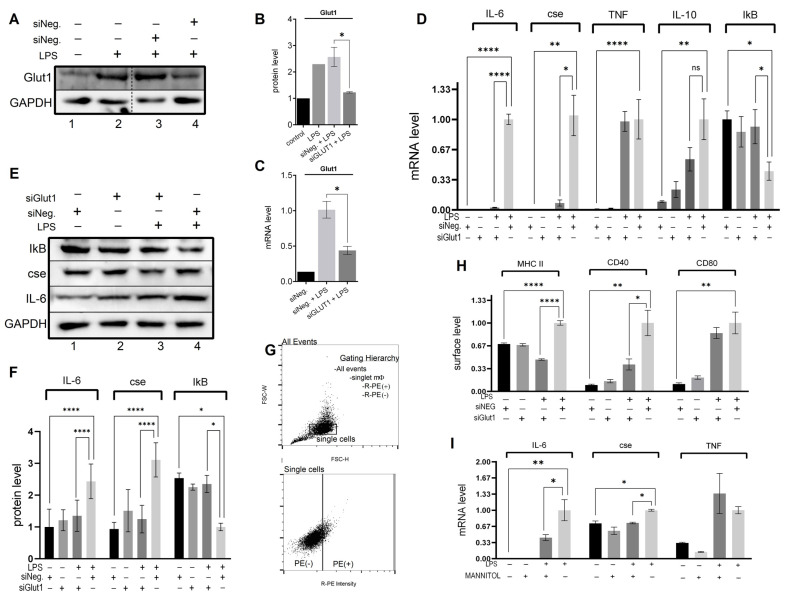
Glut1-derived glucose supports LPS-induced cytokine expression and inflammatory mediator expression (**A**–**H**). RAW264.7 silenced of Glut1 using siRNAs for 24 h. Cells were treated with LPS (100 ng/mL) for 4 h, and protein was extracted from macrophages and analyzed by Western blot using antibodies against Glut1, CSE, IL-6, IkB, and GAPDH (loading control) (**A**,**E**). Quantifications (using ImageJ software) are shown in panels (**B**,**F**). Total RNA was isolated, reverse transcribed to cDNA, and RT-qPCR analyzed the expression of CSE, IL-6, IL-10, TNFa, and IkB. GAPDH was used as a control. The expression (relative to GAPDH) of mRNA is shown in panel (**D**). For flow cytometry, a gating scheme followed a hierarchy that was selected for single cells (non-aggregate) followed by rectangle gates set based upon the isotype controls in panel (**G**). The surface expression of MHC II, CD40, and CD80 was determined following 24 h LPS stimulation; Quantifications of flow cytometry data are summarized in panel (**H**). The levels of IL-6, CSE, and TNFa mRNA were analyzed in RAW cells pretreated with low glucose (1 g/mL) DMEM supplemented with either normal (added 3 g/mL glucose) or mannitol (osmolarity control; added 3 g/mL) for 24  h followed by stimulation with LPS for 4 h (**I**). Each experiment was repeated at least with three parallel replicates. Data represent mean  ±  SD (*n*  =  3); * *p* < 0.05, ** *p*  <  0.001, **** *p* < 0.00001.

Here, to investigate if Glut1 plays any role in regulating LPS-induced pro-inflammatory response, we knocked down Glut1 (using siRNAs), then treated it with LPS, and analyzed the effect on inflammatory cytokines, CSE gene and IkB mRNA and protein expressions and MHC II surface expression. Briefly, RAW264.7 macrophages were transfected with Glut1 or negative siRNAs (24 h), followed by treatment with LPS (4 h). RNA and proteins were isolated and analyzed by RT-qPCR and Western blot. The expression of Glut1 is significantly decreased at the protein (*p* = 0.02) and mRNA (*p* = 0.01) levels upon application of Glut1 siRNA (Figure 1A–C). The effect of siGlut1 on IL-6, CSE, TNF, IL-10, and IkB mRNA expression was then determined by RT-qPCR. Analysis showed that LPS-induced IL-6 (mRNA level), cse, TNF, and IL-10 are significantly increased (siNeg. vs. siNeg. + LPS) (*p* < 0.001, *p* = 0.009, *p* < 0.001, *p* = 0.015, respectively), and IkB decreased (*p* = 0.014). Upon Glut1-knockdown the level of IL-6 and CSE are significantly decreased (*p* < 0.001, *p* = 0.0128, respectively) (Figure 1D). Western blot analysis also confirmed that LPS-induced IL-6 and CSE protein levels significantly increased (both *p* < 0.001) and decreased upon Glut1-knockdown (both *p* < 0.001), and IkB is significantly increased (*p* = 0.02) (Glut1-siRNA treatment, Figure 1E, quantification in 3F). For immunofluorescence, following siRNA treatments (24 h) and LPS (24 h), cells were removed from culture plates and fixed in suspension for MHC II, CD40, and CD80 surface staining. For flow cytometry, a gating scheme followed a hierarchy that selected for single cells (non-aggregate) followed by a polygon gate that excluded autofluorescence was used (Figure 1G). Immunostaining analysis showed a significant increase in MHC II (*p* < 0.001), CD40 (*p* = 0.001), and CD80 (*p* < 0.001) levels following LPS stimulation, and MHCII and CD40 were decreased following siGlut1 treatment (*p* < 0.001 and *p* = 0.0131, respectively) (Figure 1H). To elucidate the correlation between glucose-induced glucose metabolism under LPS stimulation and the expression of IL-6 CSE and TNF, we observed the gene expression of groups with normal glucose versus low glucose (mannitol serving as the osmolarity control for low glucose samples). Our analysis revealed significantly reduced levels of IL-6 (*p* = 0.031) and CSE (*p* = 0.018), but not TNF mRNA in LPS-treated groups comparing low glucose (1 g/mL glucose + 3 mg/mL mannitol) and normal glucose (4 g/mL glucose) (Figure 1I). These results demonstrate that Glut1 activity drives pro-inflammatory response associated with NF-κB activity that promotes inflammatory cytokine IL-6 and gasomediator H_2_S-producing enzyme CSE and supports antigen presentation and co-stimulatory activities via MHCII and CD40 surface expression. However, TNF and IL-10 mRNA and CD80 surface levels were unchanged following Glut1 silencing.

### 3.2. Glut1-Driven Glucose Uptake Alters Redox in Macrophages

**Figure 2:**
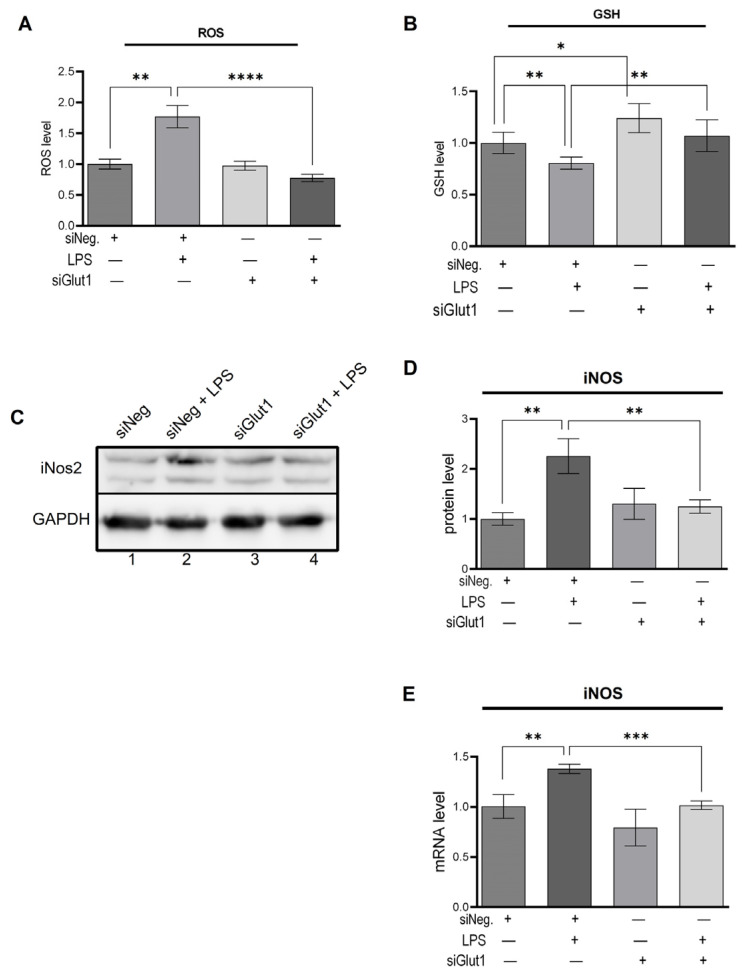
Silencing Glut1 inhibits ROS production and increased GSH level in LPS-stimulated macrophages (**A**–**E**) RAW264.7 cells were silenced of Glut1 using siRNAs for 24 h, then treated with LPS (100 ng/mL) for 4 h. ROS level was detected using a fluorometric kit and is shown in panel (**A**). ROS experiment was done for four parallel replicates. GSH level was detected using a fluorometric kit and is shown in panel (**B**). GSH experiment was done for five parallel replicates. Protein was extracted from macrophages and analyzed by Western blot using antibodies against iNOS and GAPDH (loading control) (**C**). Quantifications (using ImageJ software) are shown in panels (**D**). Total RNA was isolated, reverse transcribed to cDNA, and RT-qPCR analyzed the expression of iNOS. GAPDH was used as a control. The expression (relative to GAPDH) of mRNA is shown in panel (**E**). Data represent mean  ±  SD (*n*  =  3); * *p* < 0.05, ** *p*  <  0.001, *** *p* < 0.0001, **** *p* < 0.00001.

To investigate the role Glut1 plays in regulating LPS-induced pro-inflammatory ROS, we knocked down Glut1 (using siRNAs), then treated it with LPS, and analyzed the effect on ROS and GSH levels, as well as iNOS protein and mRNA expression. The level of ROS was significantly increased following LPS treatment (*p* < 0.001), which was reversed significantly with the application of siGlut1 (vs. siNeg + LPS group, *p* < 0.001) (Figure 2A). GSH levels are an indicator of antioxidant status, which were significantly decreased following LPS treatment (*p* = 0.003) (Figure 2B). However, without LPS application, siGlut1 significantly increased GSH levels above siNeg control (*p* = 0.007). The application of siGlut1 in LPS-treated groups ameliorated GSH levels (*p* = 0.004). Western blot analysis confirmed that LPS-induced iNOS protein level is significantly increased (*p* = 0.004), and upon Glut1-knockdown was decreased (*p* = 0.009) (Figure 2C, quantification in 2D). The iNOS mRNA expression is significantly increased (*p* = 0.007), and upon Glut1-knockdown was decreased (*p* < 0.001) (Figure 2E). These results demonstrate the important role of Glut1-derived glucose in regulating macrophage redox levels during inflammation, in addition to influencing GSH levels without immune stimulation.

### 3.3. Glut1 Silencing Increases Autophagy in Macrophages

**Figure 3:**
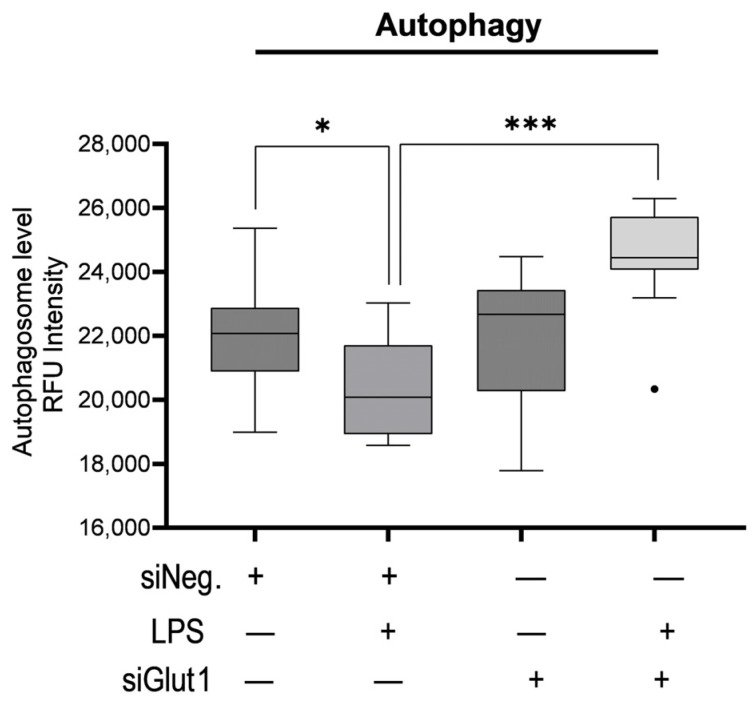
Silencing Glut1 increased autophagy in LPS-stimulated macrophages. RAW264.7 cells were silenced of Glut1 using siRNAs for 24 h, then treated with LPS (100 ng/mL) for 12 h. Autophagy was then detected in a 96-well plate format with a fluorometric kit and read by a microplate reader EX/EM = 355/538 nm. Data represent mean  ±  SD (*n*  =  14); * *p* < 0.05, *** *p* < 0.0001.

To investigate the role Glut1 plays in regulating autophagy with or without immune stimulation, we knocked down Glut1 (using siRNAs), then treated it with LPS (12 h), and analyzed the effect on autophagosome formation. Autophagy was significantly decreased following LPS treatment in siNeg-treated cells (*p* = 012). However, the application of siGlut1 significantly increased autophagy over siNeg in LPS-treated cells (*p* < 0.001) (Figure 3). Thus, it is apparent that Glut1 activity plays a role in autophagosome formation in macrophages during immune stimulation.

### 3.4. Silencing of Glut1 Triggers Apoptotic Signaling in Macrophage

**Figure 4:**
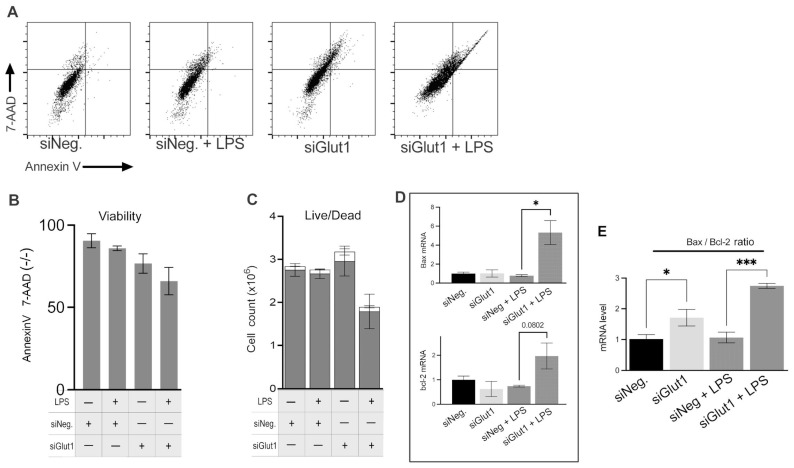
Silencing of Glut1 increased apoptosis in LPS-treated macrophages (**A**–**C**) RAW264.7 cells were silenced of Glut1 using siRNAs for 24 h, then treated with LPS (100 ng/mL) for 4 h. For apoptosis analysis, annexin V surface stain and 7-AAD DNA stain dyes were added to samples and read by flow cytometry (**A**). Quantification of Annexin V and 7-AAD negative (live) cells is shown in panel (**B**). Hemacytometer cell counts of trypan blue stained cells (live/dead discrimination) (live cells grey bars, and dead cells empty bars) are shown in panel (**C**). Total RNA was isolated, reverse transcribed to cDNA, and RT-qPCR analyzed the expression of bax and bcl-2 is shown in panel (**D**), and was also reported as the ratio of bax/bcl-2 (**E**). GAPDH was used as a control. Data represent mean  ±  SD (*n*  =  3); * *p* < 0.05, *** *p*  <  0.0001.

Macrophages are glucose-dependent, especially when initiating proinflammatory responses. We wondered whether decreased intracellular glucose alters cell viability during LPS stimulation in macrophages. To investigate the role Glut1 plays in regulating apoptosis and apoptosis signaling in macrophages, we silenced Glut1 and analyzed its effect vs. negative siRNAs with or without LPS stimulation on Annexin V and 7AAD stains (Figure 4A,B). We also investigated the bax and bcl-2 mRNA expression ratio to suggest intrinsic pathway apoptosis signaling (Figure 4C). Annexin V (AV) levels are a marker of apoptosis on the outer leaflet of the plasma membrane, and 7-AAD binds strongly to DNA and indicates the levels of dead cells. For flow cytometry, a gating scheme followed a hierarchy that excluded debris particulates using a live cell control, followed by a quadrant gate that excluded autofluorescence and background stain was used (Figure 4A). The number of live cells (AV and 7-AAD negative) was unchanged with or without LPS treatment in siNeg groups. With the application of siGlut1, live cells were slightly decreased (compare siNeg *p* = 0.17); however, with LPS and Glut1 silencing, viability was significantly decreased vs siNeg + LPS (*p* = 0.0099) (Figure 4B). Hemacytometer counted total cell number was decreased in siGlut1 + LPS group (Figure 4C). RT-qPCR determined the effect of siGlut1 on bax and bcl-2 mRNA ratio expression. Bax and bcl-2 mRNAs were measured by qPCR and the bax mRNA increased significantly compared to LPS vs. siNeg + LPS (*p* = 0.0111). The ratio of the mRNA levels of bax/bcl-2 from Figure 4D is reported in Figure 4E. Analysis showed that bax/bcl-2 is significantly increased upon Glut1-silencing in LPS-treated and untreated groups vs. the appropriate controls (*p* = 0.03; vs. *p* = 0.004, respectively) (Figure 4D). Thus, macrophages deprived of Glut1-derived glucose negatively impact cell viability and mitochondrial intrinsic pathway signaling.

## 4. Discussion

Inflammation is a physiological and regulated response to injury and infection to regain homeostasis by producing pro- and anti-inflammatory mediators. Macrophages are a major component of the mononuclear phagocyte system that participate actively in inflammation by phagocytosis, antigen presentation, and modulating immune response through the production of various inflammatory mediators [15]. Glut1 is the major glucose transporter in macrophages, and glucose substrate is important for their survival and activity [2,7]. In this study, we observed that silencing the Glut1 gene by siRNA altered the pro-inflammatory profile, redox status, apoptosis, and autophagy in LPS-activated macrophages.

LPS induced the increased mRNA and protein expression of various cytokines and inflammatory markers, including IL-6, IL-10, TNFα, iNOS, and surface markers MHC II, CD40, and CD80. Silencing Glut1 resulted in the attenuation of IL-6 and iNOS at the mRNA and protein levels. In addition, LPS-induced MHC II and CD40 surface levels were reduced following Glut1 knockdown. Interestingly, we also observed that Glut1 is necessary for LPS-induced CSE gene expression, indicating its involvement in the transsulfuration pathway. To confirm the relationship between glucose-induced glucose metabolism and the expression of IL-6, CSE, and TNF under LPS stimulation, we compared gene expression in macrophages cultured in normal glucose and low glucose (with mannitol as the osmolarity control). We demonstrate that glucose plays a direct role in regulating the expression of IL-6 and CSE and maintaining surface levels of MHCII and CD40 within these cells, highlighting the importance of glucose metabolism in maintaining the normal immune-modulatory, antigen-presentation, and co-stimulatory function of macrophages during inflammation.

Freemerman et al. (2019) studied the effect of CRISPR/Cas9-mediated Glut1 knockout on IL-6, IL-10, and TNF production in bone marrow-derived macrophages challenged with LPS and IFN-γ [16]. Unlike our study, they found no impact on IL-6 levels but observed similar effects on TNF and IL-10 expression. Differences between the studies could be due to compensatory mechanisms or a more complex microenvironment in the in vivo model, emphasizing the nuanced interplay between these systems in macrophages.

NF-κB regulates the transcription of many pro-inflammatory cytokines and chemokines. In unstimulated conditions, it is bound to an inhibitory protein kB (IkBα) in the cytoplasm. Upon activation, NF-κB dissociates from IkBα and translocates into the nucleus to induce the expression of inflammatory genes [17]. Mechanistically, we associate NF-κB activity with this inflammatory profile. Indeed, silencing Glut1 in LPS-activated macrophages stabalized IkBα protein and mRNA following LPS treatment and consequently decreased inflammatory mediator levels, consistent with previous studies [8,11]. Previously it was shown by our group that inhibition of H_2_S production via silencing the CSE gene in macrophages results in decreased NF-κB activity [9] and Glut1 expression [18]. These data show the unusual cross-regulation between glucose utilization and H_2_S production in inflammatory macrophage via NF-κB. Glut1-induced glycolytic burst induces the formation of pro-inflammatory ROS, which is important to activate NF-κB [16] and fight pathogens.

CSE is the major source of H_2_S and cysteine in macrophages, and CSE upregulation indicates increased production of both of these metabolites. CSE-derived cysteine controls GSH levels in cells [19], which is a critical antioxidant in cells [20,21,22]. However, H_2_S is an inflammatory mediator and significantly increased levels of this gaseous molecule are reported as a contributor in various acute [23,24] and chronic [25] inflammatory diseases. In physiological concentration, H_2_S may regulate glucose metabolism in macrophages and stimulate antioxidant gene transcription [26]. A higher concentration of H_2_S has cytotoxic impacts via the inhibition of mitochondrial cytochrome C oxidase [27,28]. H_2_S either endogenously Click or tap here to enter text.produced or from exogenous sources appears to create a favorable redox milieu for glycolysis [27,28,29], suggesting potential crosstalk. Nevertheless, the precise effect of H_2_S on glut1 regulation and its controllers remains elusive and requires additional studies to elucidate.

Glut1-derived glucose utilization is critical for ROS formation in LPS-stimulated macrophages. This pro-inflammatory response requires macrophages to induce cell processes that allow protection from the aggressive local environment, such as increased GSH production. During immune response in macrophages, CSE-derived H_2_S is increased and acts as an immune signaling molecule that may enhance cellular antioxidant status [26,30] and participates in immune signaling [23,24,25]. To elucidate the indirect effect upon ROS and iNOS as well as GSH in association with Glut1 following Glut1 silencing, we measured the ROS and GSH levels and iNOS mRNA and protein levels. First, we confirm that Glut1 is critical for LPS-induced ROS formation and iNOS mRNA and protein expression. Loss of Glut1 inhibited LPS-stimulated pro-oxidant and anti-microbial potential in macrophages by inhibiting glucose-derived ROS and decreased the transcription of NO synthesizing iNOS. The loss of glucose-derived ROS and NO ameliorated LPS-induced oxidative stress, evidenced by the restored GSH levels following Glut1 silencing.

Phagocytosis, autophagy, lysosomal fusion, and antigen presentation through the MHC II system are critical for activating the adaptive immune system and play key roles in the progression of the immune response [31]. Here, we show that with LPS stimulation, autophagy levels were decreased in macrophages, which is consistent with previous reports [32]. Autophagy activity within macrophages is received continuously [33] and is associated with delivering cytosolic proteins for MHC II presentation to improve helper T cell stimulation [5]. Autophagosome formation abrogates pro-inflammatory cytokine secretion and plays a role in regulating immune endocrine signaling [34], and regulates NLRP3 inflammasome inflammation [35]. Enhanced autophagosome formation in LPS-treated Glut1 silenced cells suggests a reduced inflammatory response of macrophages. These results are consistent with a previous report showing that high glucose-treated macrophages show decreased levels of autophagy [35]. It is yet unresolved why autophagy is increased in these cells and future work is required to clarify the mechanism regulating this observation. It is possible that autophagy can help in the clearance of damaged mitochondria, which are a significant source of ROS. By removing damaged mitochondria, autophagy can reduce ROS production, thus mitigating oxidative stress and the resulting inflammatory response. In addition, the absence of glucose uptake, cells may rely on alternative metabolic pathways and increased autophagy, potentially influenced by LPS, to generate energy and maintain homeostasis; however, further experiments are needed for confirmation.

Here, Glut1 silencing leads to a partial switch in macrophage phenotype. The significant decrease in IL-6, ROS, and iNOS, which are primarily associated with M1 activation, indicates a shift towards a less pro-inflammatory state. The maintenance of IL-10 levels, an anti-inflammatory cytokine, supports this interpretation. However, the unchanged TNF-α levels (LPS vs. siGlut1 + LPS) suggest that some aspects of the M1 phenotype are still maintained. TNF-α is a pro-inflammatory cytokine, and its presence indicates that the macrophages may not have fully transitioned to an M2 phenotype. These findings suggest that Glut1 silencing might lead to a mixed or intermediate macrophage phenotype rather than a complete switch from M1 to M2. This highlights the complexity and plasticity of macrophage polarization, which can be influenced by various factors such as metabolic pathways, including Glut1-mediated glucose metabolism. Further studies could explore the functional consequences of this partial phenotypic switch and the molecular mechanisms involved in this process.

It is well known that macrophages are glucose-dependent. Indeed, LPS treatment of Glut1 silenced macrophages elevated levels of annexin V release on the outer leaflet of the plasma membrane and levels of dead cells, indicating increased activation of apoptosis. The Bax/Bcl-2 Ratio determines the susceptibility of cells to CD95/Fas-mediated intrinsic pathway apoptosis [36]. Indeed, increased intrinsic pathway apoptosis signaling pathways (Bax/Bcl-2 ratio) were observed in Glut1 silenced cells with and without the addition of LPS, suggesting the crucial role of Glut1 in maintaining homeostatic energy conditions that influence not only macrophage inflammatory response but also survival.

The major conclusion of the present study is that inhibition of inflammation-induced metabolic reprogramming by silencing Glut1 using siRNAs impairs the pro-inflammatory activity of macrophages. Inhibition of glucose uptake during immune stimulation is associated with decreased activation of NF-κB and the reduced production of IL-6, ROS, iNOS, MHC II and CD40 surface levels, and CSE, which correlates with H_2_S production. Silencing Glut1 in LPS-stimulated macrophages increases autophagosome formation. Targeting Glut1 could potentially serve as a therapeutic strategy to control excessive inflammation in diseases like atherosclerosis or sepsis. For future studies, it is necessary to validate the findings of this study using primary cells from animal or human samples, as they may provide a more accurate representation of the in vivo situation. Preliminary cell line studies are vital for understanding basic biology and mechanisms. Our results suggest that Glut1-targeted glucose flux could be a potential therapy to curb macrophage inflammatory phenotype and serve as a target in inflammatory diseases. Further investigation is required to determine Glut1’s anti-inflammatory capabilities.

## Figures and Tables

**Table 1:** PCR primer sequences.

Gene	Forward (5′–3′)	Reverse (5′–3′)
*Glut1*	GATCTGAGCTACGGGGTCTT	TGTAGAACTCCTCAATAACCTTCTG
*IL-6*	CGGCCTTCCCTACTTCACAA	TTGCCATTGCACAACTCTTTTC
*TNFα*	CCCACGTCGTAGCAAACCAC	TAGCAAATCGGCTGACGGTG
*IL-10*	GGTTGCCAAGCCTTATCGGA	CACCTTGGTCTTGGAGCTTATT
*iNOS2*	GGTGAAGGGACTGAGCTGTT	ACGTTCTCCGTTCTCTTGCAG
*IkBα*	ACAACAGAGATGAGGGCGATG	CTCTGGAGGGTCGGGACTTA
*CSE*	CAAAGCAACACCTCGCACTC	ATGCAAAGGCCAAACTGTGC
*Bcl-2*	CTGAGTACCTGAACCGGCAT	AGTTCCACAAAGGCATCCCAG
*Bax*	TGGAGCTGCAGAGGATGATT	TCTTGGATCCAGACAAGCAGC
*GAPDH*	CGTCCCGTAGACAAAATGGT	GAGGTCAATGAAGGGGTC

## Data Availability

The datasets generated for this study are available on Mendelay Data, at reserved Doi: 10.17632/wp95sn56fs.1.

## References

[1] Wang N., Liang H., Zen K. (2014). Molecular Mechanisms That Influence the Macrophage M1-M2 Polarization Balance. Front. Immunol..

[2] Freemerman A.J., Johnson A.R., Sacks G.N., Milner J.J., Kirk E.L., Troester M.A., Macintyre A.N., Goraksha-Hicks P., Rathmell J.C., Makowski L. (2014). Metabolic Reprogramming of Macrophages: Glucose Transporter 1 (GLUT1)-Mediated Glucose Metabolism Drives a Proinflammatory Phenotype. J. Biol. Chem..

[3] Jakubzick C.V., Randolph G.J., Henson P.M. (2017). Monocyte Differentiation and Antigen-Presenting Functions. Nat. Rev. Immunol..

[4] Hirayama D., Iida T., Nakase H. (2018). The Phagocytic Function of Macrophage-Enforcing Innate Immunity and Tissue Homeostasis. Int. J. Mol. Sci..

[5] Schmid D., Münz C. (2007). Innate and Adaptive Immunity through Autophagy. Immunity.

[6] Tannahill G.M., Curtis A.M., Adamik J., Palsson-Mcdermott E.M., McGettrick A.F., Goel G., Frezza C., Bernard N.J., Kelly B., Foley N.H. (2013). Succinate Is an Inflammatory Signal That Induces IL-1β through HIF-1α. Nature.

[7] Obaid M., Udden S.M.N., Alluri P., Mandal S.S. (2021). LncRNA HOTAIR Regulates Glucose Transporter Glut1 Expression and Glucose Uptake in Macrophages during Inflammation. Sci. Rep..

[8] Badiei A., Gieseg S., Davies S., Othman M.I., Bhatia M. (2015). LPS Up-Regulates Cystathionine γ -Lyase Gene Expression in Primary Human Macrophages via NF-ΚB/ERK Pathway. Inflamm. Allergy-Drug Targets.

[9] Badiei A., Muniraj N., Chambers S., Bhatia M. (2014). Inhibition of Hydrogen Sulfide Production by Gene Silencing Attenuates Inflammatory Activity by Downregulation of NF-ΚB and MAP Kinase Activity in LPS-Activated RAW 264.7 Cells. Biomed. Res. Int..

[10] Whiteman M., Li L., Rose P., Tan C.H., Parkinson D.B., Moore P.K. (2010). The Effect of Hydrogen Sulfide Donors on Lipopolysaccharide-Induced Formation of Inflammatory Mediators in Macrophages. Antioxid. Redox. Signal..

[11] Zheng Y., Luo N., Mu D., Jiang P., Liu R., Sun H., Xiong S., Liu X., Wang L., Chu Y. (2013). Lipopolysaccharide Regulates Biosynthesis of Cystathionine γ-Lyase and Hydrogen Sulfide through Toll-like Receptor-4/P38 and Toll-like Receptor-4/NF-ΚB Pathways in Macrophages. Vitr. Cell. Dev. Biol.-Anim..

[12] Smith P.K., Krohn R.I., Hermanson G.T., Mallia A.K., Gartner F.H., Provenzano M.D., Fujimoto E.K., Goeke N.M., Olson B.J., Klenk D.C. (1985). Measurement of Protein Using Bicinchoninic Acid. Anal. Biochem..

[13] Taylor S.C., Nadeau K., Abbasi M., Lachance C., Nguyen M., Fenrich J. (2019). The Ultimate QPCR Experiment: Producing Publication Quality, Reproducible Data the First Time. Trends. Biotechnol..

[14] Rahman I., Kode A., Biswas S.K. (2007). Assay for Quantitative Determination of Glutathione and Glutathione Disulfide Levels Using Enzymatic Recycling Method. Nat. Protoc..

[15] Fujiwara N., Kobayashi K. (2005). Macrophages in Inflammation. Curr. Drug Targets-Inflamm. Allergy.

[16] Freemerman A.J., Zhao L., Pingili A.K., Teng B., Cozzo A.J., Fuller A.M., Johnson A.R., Milner J.J., Lim M.F., Galanko J.A. (2019). Myeloid Slc2a1-Deficient Murine Model Revealed Macrophage Activation and Metabolic Phenotype Are Fueled by GLUT1. J. Immunol..

[17] Karin M., Ben-Neriah Y. (2003). Phosphorylation Meets Ubiquitination: The Control of NF-ΚB Activity. Annu. Rev. Immunol..

[18] Cornwell A., Fedotova S., Cowan S., Badiei A. (2022). Cystathionine γ-Lyase and Hydrogen Sulfide Modulates Glucose Transporter Glut1 Expression via NF-ΚB and PI3k/Akt in Macrophages during Inflammation. PLoS ONE.

[19] Lee Z.W., Low Y.L., Huang S., Wang T., Deng L.W. (2014). The Cystathionine γ-Lyase/Hydrogen Sulfide System Maintains Cellular Glutathione Status. Biochem. J..

[20] Sbodio J.I., Snyder S.H., Paul B.D. (2019). Regulators of the Transsulfuration Pathway. Br. J. Pharmacol..

[21] McBean G.J., Aslan M., Griffiths H.R., Torrão R.C. (2015). Thiol Redox Homeostasis in Neurodegenerative Disease. Redox. Biol..

[22] Paul B.D., Sbodio J.I., Snyder S.H. (2018). Cysteine Metabolism in Neuronal Redox Homeostasis. Trends Pharmacol. Sci..

[23] Badiei A., Chambers S.T., Gaddam R.R., Bhatia M. (2016). Cystathionine-γ-Lyase Gene Silencing with SiRNA in Monocytes/Macrophages Attenuates Inflammation in Cecal Ligation and Puncture-Induced Sepsis in the Mouse. J. Biosci..

[24] Gaddam R.R., Fraser R., Badiei A., Chambers S., Cogger V.C., Le Couteur D.G., Ishii I., Bhatia M. (2016). Cystathionine-Gamma-Lyase Gene Deletion Protects Mice against Inflammation and Liver Sieve Injury Following Polymicrobial Sepsis. PLoS ONE.

[25] Muniraj N., Stamp L.K., Badiei A., Hegde A., Cameron V., Bhatia M. (2017). Hydrogen Sulfide Acts as a Pro-Inflammatory Mediator in Rheumatic Disease. Int. J. Rheum. Dis..

[26] Lohninger L., Tomasova L., Praschberger M., Hintersteininger M., Erker T., Gmeiner B.M.K., Laggner H. (2015). Hydrogen Sulphide Induces HIF-1α and Nrf2 in THP-1 Macrophages. Biochimie.

[27] Libiad M., Vitvitsky V., Bostelaar T., Bak D.W., Lee H.J., Sakamoto N., Fearon E., Lyssiotis C.A., Weerapana E., Banerjee R. (2019). Hydrogen Sulfide Perturbs Mitochondrial Bioenergetics and Triggers Metabolic Reprogramming in Colon Cells. J. Biol. Chem..

[28] Vitvitsky V., Kumar R., Libiad M., Maebius A., Landry A.P., Banerjee R. (2021). The Mitochondrial NADH Pool Is Involved in Hydrogen Sulfide Signaling and Stimulation of Aerobic Glycolysis. J. Biol. Chem..

[29] Cornwell A., Badiei A. (2023). From Gasotransmitter to Immunomodulator: The Emerging Role of Hydrogen Sulfide in Macrophage Biology. Antioxidants.

[30] Calvert J.W., Jha S., Gundewar S., Elrod J.W., Ramachandran A., Pattillo C.B., Kevil C.G., Lefer D.J. (2009). Hydrogen Sulfide Mediates Cardioprotection through Nrf2 Signaling. Circ. Res..

[31] Allavena P., Sica A., Solinas G., Porta C., Mantovani A. (2008). The Inflammatory Micro-Environment in Tumor Progression: The Role of Tumor-Associated Macrophages. Crit. Rev. Oncol. Hematol..

[32] Cao Y., Chen J., Ren G., Zhang Y., Tan X., Yang L. (2019). Punicalagin Prevents Inflammation in LPS- Induced RAW264.7 Macrophages by Inhibiting FoxO3a/Autophagy Signaling Pathway. Nutrients.

[33] Schmid D., Pypaert M., Münz C. (2007). Antigen-Loading Compartments for Major Histocompatibility Complex Class II Molecules Continuously Receive Input from Autophagosomes. Immunity.

[34] Ying Y., Sun C.-B., Zhang S.-Q., Chen B.J., Yu J.Z., Liu F.Y., Wen J., Hou J., Han S.S., Yan J.Y. (2021). Induction of Autophagy via the TLR4/NF-ΚB Signaling Pathway by Astragaloside IV Contributes to the Amelioration of Inflammation in RAW264.7 Cells. Biomed. Pharmacother..

[35] Xue Z., Zhang Z., Liu H., Li W., Guo X., Zhang Z., Liu Y., Jia L., Li Y., Ren Y. (2019). LincRNA-Cox2 Regulates NLRP3 Inflammasome and Autophagy Mediated Neuroinflammation. Cell Death Differ..

[36] Raisova M., Hossini A.M., Eberle J., Riebeling C., Wieder T., Sturm I., Daniel P.T., Orfanos C.E., Geilen C.C. (2001). The Bax/Bcl-2 Ratio Determines the Susceptibility of Human Melanoma Cells to CD95/Fas-Mediated Apoptosis. J. Investig. Dermatol..

